# Identification of oligosaccharyltransferase as a host target for inhibition of SARS-CoV-2 and its variants

**DOI:** 10.1038/s41421-021-00354-2

**Published:** 2021-11-30

**Authors:** Yi-Jiao Huang, Hui Zhao, Xun Huang, Yong-Qiang Deng, Xiao-Feng Li, Qing Ye, Rui-Ting Li, Yan-Peng Xu, Tian-Shu Cao, Cheng-Feng Qin

**Affiliations:** 1 State Key Laboratory of Pathogen and Biosecurity, Beijing Institute of Microbiology and Epidemiology, Academy of Military Medical Sciences, Beijing, China; 2 Research Unit of Discovery and Tracing of Natural Focus Diseases, Chinese Academy of Medical Sciences, Beijing, China

**Keywords:** Glycobiology, Glycosylation

Dear Editor,

According to the report of WHO (https://covid19.who.int/), COVID-19, caused by the pandemic pathogen, severe acute respiratory syndrome coronavirus 2 (SARS-CoV-2), has led to over 250 million confirmed cases and at least 5 million deaths, as of November 2021. Vaccine administration is currently the most effective way to control the COVID-19 pandemic, while novel variants of SARS-CoV-2 with concerning mutations have thrived throughout the world. Many of these variants have been evidenced to enhance viral transmissibility, fitness, infectivity, and even evade protection from vaccines^1^. Thus, there is an urgent need for developing effective antiviral drugs against SARS-CoV-2. So far, a large panel of antiviral agents showed promising efficiency against SARS-CoV-2 in either preclinical studies or clinical trials^2–4^.

Targeting host proteins associated with SARS-CoV-2 represents an alternative strategy to antagonize the emerging variants. Therapies that target the host–virus interface, which is relatively conserved between reported SARS-CoV-2 strains, could present broad-spectrum antiviral potentials^5,6^. Some studies have mapped the SARS-CoV-2–host interactome^5,7^, however, it remains a challenge to systematically explore the critical host proteins that have been already or potentially targeted by drugs.

The genome of SARS-CoV-2, which is about 29.8 kb in size, comprises two flanking untranslated regions and 14 open reading frames (ORFs) that encode 27 proteins, and shares ~80% nucleotide sequence identity with SARS-CoV^8^. SARS-CoV-2 contains four structural proteins, spike (S), envelope (E), membrane (M), and nucleocapsid (N) proteins^8^. The spike, located on viral surface, is constituted by the homotrimers of S protein, and responsible for the recognition of host cell receptor(s)^9^. M protein, containing three transmembrane domains, can shape the virions and promote membrane curvature to facilitate the binding with N proteins, which bind virus RNA genome through different mechanisms^9^. E protein executes an important role in the assembly and release of virus, and is implicated in modulating viral pathogenesis^9^. Since these structural proteins play critical roles in the virion assembly and infection of SARS-CoV-2^9^, we screened the host factors that interact with these proteins. We found that the oligosaccharyltransferase (OST) complex is closely associated with E, M and S proteins, and blockade of OST by NGI-1 can significantly inhibit the infection of both SARS-CoV-2 and its variants.

To identify the potential host factors that interact with SARS-CoV-2 structural proteins, we first synthesized codon-optimized virus cDNAs of E, M, S and N proteins, and cloned these ORFs into mammalian expression vectors. The expressed structural proteins contain an N-terminal 3× Flag tag and a C-terminal Twin-Strep-tag. We next expressed these structural proteins in HEK293T, and screened the interacting host proteins by affinity-purification-mass spectrometry (AP-MS). We found that E, M and S proteins can associate with many N-Glycosylation-related proteins, especially the OST complex, which can catalyze the N-linked glycosylation of newly synthesized proteins^10^ (Fig. 1a). In addition to E, M, S and N proteins, we also detected the interacting proteins of another SARS-CoV-2-derived protein, 3a, which has been reported as a potential structural glycoprotein in SARS-CoV^11^, similar to SARS-CoV-2 M protein. Several N-Glycosylation-related proteins can interact with 3a (Fig. 1a). These data suggested that E, M and S proteins may be N-Glycosylated in host cells. To examine this hypothesis, we treated the pull-down samples with PNGase F, an amidase that can remove N-linked oligosaccharides from glycoproteins. As shown in Fig. 1b, after PNGase F treatment, the major spike band shifted to lower than 180 kDa (Fig. 1b), indicating that spike is heavily N-Glycosylated, which is consistent with other studies^12,13^. Different from S protein, both E and M proteins displayed broad bands and main bands. After PNGase F treatment, the broad bands were decreased, while the main bands of E and M (~20 kDa and ~30 kDa, respectively) were increased (Fig. 1b), suggesting that both E and M proteins may be glycosylated, while other post-translational modification or oligomerization may also occur. There was no obvious variation observed for 3a and N proteins after PNGase F treatment (Supplementary Fig. S1a). These results indicated that E, M and S proteins were N-Glycosylated, and OST complex may play an important role in mediating this post-translational modification.

**Fig. 1:**
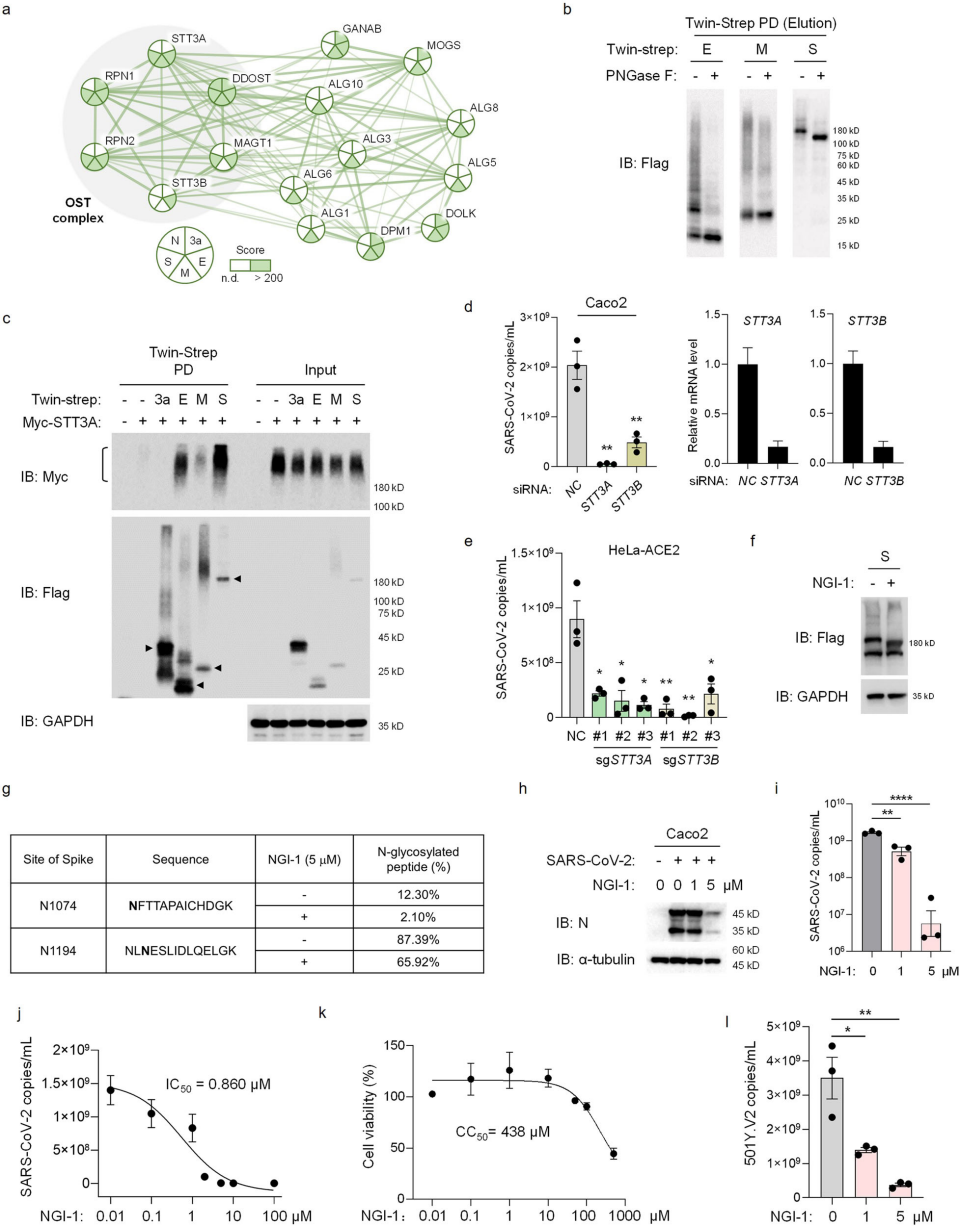
NGI-1 inhibited SARS-CoV-2 by OST. **a** Biological network cluster of N-linked glycosylation according to AP-MS analysis. The original score of identified host proteins interacting with viral proteins was indicated by color scale within the circle. Score > 200 indicated sufficient confidence. n.d., not detected. The pie chart was divided into 5 slices, representing 3a, E, M, S and N proteins of SARS-CoV-2, respectively. The network was delivered according to STRING website (https://string-db.org/). Network in the gray shaded area showed OST complex. **b** 3× Flag-Twin-Strep tagged E, M or S proteins of SARS-CoV-2 were overexpressed in HEK293T. Proteins were pulled down after ultrasonication and eluted for further PNGase F treatment. Immunoblot analysis of virus proteins with Flag antibody. **c** Myc-STT3A and 3× Flag-Twin-Strep tagged 3a, E, M or S proteins of SARS-CoV-2 were overexpressed in HEK293T cells, followed by Twin-Strep pull-down. STT3A was analyzed with Myc antibody and viral proteins were analyzed with Flag antibody. The anti-GAPDH blots indicated loading of lanes. **d** STT3A or STT3B was knocked down in Caco2 cells for 3 days, followed by 3-day-SARS-CoV-2 infection (MOI = 0.01). The supernatant was collected to detect the SARS-CoV-2 gRNA by real-time quantitative polymerase chain reaction (RT-qPCR). The cells were collected to detect mRNA levels of *STT3A* or *STT3B* by quantitative polymerase chain reaction (qPCR) (*GAPDH* as a reference gene). NC, negative control. **e** STT3A or STT3B was knocked out by 3 different sgRNAs respectively in HeLa cells stably expressing human ACE2 (HeLa-ACE2). After 3-day-infection, SARS-CoV-2 in the supernatant was analyzed by RT-qPCR. **f**, **g** 3× Flag-tagged spike of SARS-CoV-2 was overexpressed in HEK293T cells, followed by 5 μM NGI-1 treatment for 2 days. Whole cell lysates were collected for Immunoblot analysis (**f**). Spike proteins were pulled down after ultrasonication and eluted for further MS (**g**). Spike was analyzed with Flag antibody. The anti-GAPDH blots indicated loading of lanes. The percentage of spike of the indicated sites, N1074 and N1194, was analyzed by Thermo Scientific Q Exactive™ Hybrid Quadrupole-Orbitrap™ Mass Spectrometer. Data are means ± SD, from triplicates (technical replicates). **h**, **i** Caco2 cells were infected with SARS-CoV-2 (MOI = 0.01) for 1 h, and the medium was replaced by 2% FBS (v/v) DMEM containing 0, 1, or 5 μM NGI-1 for further 3-day treatment. SARS-CoV-2 in the supernatant was analyzed by RT-qPCR (**i**). Virus in cells was detected by immunoblotting with N protein antibody (**h**). The anti-α-tubulin blots indicated loading of lanes (**h**). **j** Caco2 cells were infected with SARS-CoV-2 (MOI = 0.01) for 1 h, followed by treatment of NGI-1 at the indicated concentrations for 3 days. Virus in the supernatant was analyzed by RT-qPCR. The IC_50_ was calculated by Statistical Package for the Social Sciences (SPSS) and the dose-response curve was fitted by GraghPad Prism 8. **k** Caco2 cells were plated in a 96-well plate and cultured in the presence of NGI-1 at indicated concentrations for 3 days. The cytotoxicity was analyzed by CellTiter 96 ^®^ AQueous One Solution Cell Proliferation Assay. The CC_50_ was calculated by SPSS and the dose-response curve was fitted by GraghPad Prism 8. **l** Caco2 cells were infected with the South Africa variant of SARS-CoV-2 501Y.V2 (MOI = 0.01) for 1 h, followed by treatment of NGI-1 at the indicated concentrations for 3 days. Virus in the supernatant was analyzed by RT-qPCR. Data are means ± SEM, from triplicates (biological replicates), unpaired *t*-test, *P < 0.05, **P < 0.01, ****P < 0.0001 (**d**, **e**, **i**–**l**).

There are two OST protein isoforms in mammalian cells^10,14^. The two isoforms are composed of a catalytic subunit (encoded by the paralogs *STT3A* or *STT3B*) and accessory subunits^14^. We then examined the interaction between the structural proteins and STT3A/STT3B by pull-down assay. Among all tested proteins, three N-Glycosylated proteins, E, M and S proteins, showed strong interaction with both STT3A and STT3B (Fig. 1c; Supplementary Fig. S1b). Blockade of either STT3A or STT3B by siRNA or CRISPR-Cas9 sgRNA suppressed SARS-CoV-2 infection (Fig. 1d, e). These results indicated that both OST isoforms could modulate functions of E, M and S proteins, and thus are critical for SARS-CoV-2 infection.

We next sought to confirm whether OST can serve as a potential target for treating SARS-CoV-2 infection. NGI-1, a small molecule that directly targets STT3A and STT3B, is an inhibitor of N-linked glycosylation with pan-flaviviral activity^14^. We first detected whether NGI-1 affected the N-glycosylation of SARS-CoV-2 E, M and S proteins. When treated with NGI-1, S protein, which was overexpressed in HEK293T, was detected at lower molecular weight, indicating that NGI-1 decreased the N-glycosylation of spike (Fig. 1f), while E and M proteins showed no obvious decrease (Supplementary Fig. S2a). We next analyzed the modification changes of S protein with quantitative mass spectrometry. We detected two N-glycosylated sites of high abundance of spike, N1074 and N1194, and the proportion of N-glycosylated peptides of spike at N1074 site decreased from 12.30% to 2.10%, and at N1194 site decreased from 87.39% to 65.92% (Fig. 1g). These results suggested that NGI-1 could decrease the N-Glycosylation of SARS-CoV-2.

Then, we investigated the antiviral effect of NGI-1 in human colorectal adenocarcinoma Caco2 cells. After 3-day infection, we examined virus replication in both the culture medium and Caco2 cells, and found a strong inhibition effect of NGI-1 on SARS-CoV-2 (Fig. 1h, i; Supplementary Fig. S2b). The half inhibitory concentration (IC_50_) of NGI-1 and the 50% cytotoxic concentration (CC_50_) of NGI-1 to Caco2 cells, were 0.860 μM and 438 μM, respectively (Fig. 1j, k). The selectivity index (SI, CC_50_/IC_50_) was ~509. In addition, we detected the IC_50_ and CC_50_ in other SARS-CoV-2-sensitive cell lines, including Huh-7, HeLa-ACE2 and Vero cells (Supplementary Fig. S2c), of which SI were about 255, 10541, and 260, respectively. Further, we showed that NGI-1 could efficiently inhibit the 501Y.V2 variant of SARS-CoV-2 (Fig. 1l). NGI-1 also exhibited a significant inhibitory effect on the replication of HCoV 229E (Supplementary Fig. S3a, b). Taken together, these results indicated that the OST inhibitor NGI-1, can serve as a broad-spectrum anti-coronavirus drug.

In the present study, we focused on the potential therapies that target the host–virus interface, especially the interactions involving structural proteins of SARS-CoV-2. By AP-MS, we found that E, M and S proteins were all N-glycosylated, and were closely related to OST-related pathways in host, suggesting that OST may serve as a potential target for treating COVID-19. Indeed, we found that blockade of the catalytic subunit, STT3A or STT3B, of OST, suppressed SARS-CoV-2 infection. Consistently, an OST inhibitor, NGI-1, blocked the infections of both wild-type SARS-CoV-2 and its variant 501Y.V2 in vitro. During the preparation of the manuscript, another study in preprint also reported similar findings^15^. As NGI-1 targets OST in host cells, all SARS-CoV-2 variants are supposed to be sensitive to NGI-1. In addition, by comparing several concerned variants with original SARS-CoV-2, we found that mutations in structural proteins, especially spike, have no effect on the existing or potential N-linked glycosylation sites (sequons, (NXT/S/C; X≠P)^10^). Interestingly, the SARS-CoV-2 variant 501Y.V3 contained the T20N mutation in S protein, which may generate an additional N-glycosylation site that can be catalyzed by OST (from TRT to NRT). The biological impact of T20N mutation remains to be determined. Moreover, the appropriate SI of NGI-1 in various cell lines supports further validation in animal model and clinical trials. Finally, chemical library-based high throughput screening for other OST inhibitors should be warranted in the future.

## Supplementary information

Supplementary material
