# Supplementary material for: Identification of oligosaccharyltransferase as a host target for inhibition of SARS-CoV-2 and its variants
Source: Cell Discov. 2021 Nov 30;7:116. doi: 10.1038/s41421-021-00354-2 (PMC8630214; doi:10.1038/s41421-021-00354-2)
Supplement: Supplementary file 1 — Supplementary material [file 41421_2021_354_MOESM1_ESM.pdf]

Supplementary information

Methods and materials

Cells and viruses

HEK293T (Human embryonic kidney, ATCC), Caco2 (Human colorectal adenocarcinoma, ATCC), Huh7 (Human hepatocarcinoma, JCRB) and Vero (African green monkey kidney, ATCC) cells were cultured in DMEM (Thermo Fisher Scientific) supplemented with 10% (vol/vol) fetal bovine serum (FBS, Thermo Fisher Scientific), penicillin (100 U/mL) and streptomycin (100 μg/mL). HeLa-ACE2 (Blasticidin resistance) was a gift from Prof. Nan Song (Beijing Institute of Tropical Medicine).

Patient-derived SARS-CoV-2 isolates (BetaCoV/Beijing/IME-BJ01/2020 (131)) were passaged as previously reported[15]. SARS-CoV-2 strain, 131 (GWHACAX01000000) were isolated from COVID-19 patients in China early 2020. The variant N501Y.V2 (CSTR.16698.06.NPRC 2.062100001) were isolated from the imported patients from South Africa. Patient-derived HCoV 229E isolates were passaged in Huh7 cells, and the virus stock was aliquoted and titrated to PFU/mL in Huh7 cells by plaque assay.

For virus infection, cells were cultured in 24-well plates at a density of 2×10^5 per well for 18 hours. After 1-hour infection, the medium was replaced by 2% FBS (V/V) DMEM containing NGI-1 (HY-117383, MCE) for further 3-day treatment.

To make the STT3A or STT3B knock-out cell lines, endogenous STT3A or STT3B was deleted in HeLa-ACE2 cell line using the lentiCRISPR system. Single-guide RNA (sgRNA) sequences of STT3A or STT3B were designed by the CRISPOR online tool (http://crispor.tefor.net/).

sgRNA	Forward primer	Reverse primer
sg*STT3A* #1	CACCGCTTTCCTAAACGATCCGCG	AAACCGCGGATCGTTTAGGAAAGC
sg*STT3A* #2	CACCGACTTTAATTATCGGACTACC	AAACGGTAGTCCGATAATTAAAGTC
sg*STT3A* #3	CACCGCCTCCAATGATTCGTCCCAA	AAACTTGGGACGAATCATTGGAGGC
sg*STT3B* #1	CACCGCGCCGCCTTGTGCGCGCACT	AAACAGTGCGCGCACAAGGCGGCGC
sg*STT3B* #2	CACCGCGTCCCTCAACTCGTCCCCG	AAACCGGGGACGAGTTGAGGGACGC
sg*STT3B* #3	CACCGCCATGTTGTGCCCCGGCGG	AAACCCGCCGGGGCACAACATGGC

Pull-down assay

HEK293T cells were cultured in 10 cm dishes at a density of 2×10^6 per dish for 18 h, and were transfected with plasmids (1 μg/mL) by TurboFect™ Transfection Reagent (Thermo Fisher Scientific). 48 h later, cells were washed with PBS for 3 times and then lysed in M2 buffer supplemented with complete protease inhibitor cocktail (04693132001, Roche), followed by centrifugation at 16,000 × g for 30 min at 4°C. The supernatants were pulled down with MagStrep "type3" XT beads (2-4090-010, IBA Lifesciences) for 4 h at 4°C. The beads were washed 4 times with M2 buffer and boiled in 1×SDS-loading buffer for immunoblot analysis.

For Mass Spectrometry analysis, gels were processed by BIOMS Ltd. Peptides were performed using the Q Exactive™ Hybrid Quadrupole-Orbitrap™ Mass Spectrometer (Thermo Fisher Scientific). Raw data were analyzed by Byonic (v3.11.3) software. The database was from uniprot_human_75777_4_20210331.fasta. For quantitative MS, raw data were analyzed by Proteome Discoverer (version 2.5.0.400).

For PNGase F treatment, cells were lysed in M2 buffer supplemented with complete protease inhibitor cocktail, followed by sonication and centrifugation at 16,000 × g for 30 min at 4°C.

The supernatants were pulled down with MagStrep "type3" XT beads for 4 h at 4°C. The beads were washed 4 times with Strep-Tactin ®XT Wash Buffer (2-1003-100, IBA Lifesciences) and eluted with Strep-Tactin ®XT Elution Buffer (2-1042-025, IBA Lifesciences). The eluted proteins were detected N-glycosylation by Rapid™ PNGase F (P0710S, NEB) according to the manufacturer's protocol.

For immunoblot analysis, samples were separated by 8-20% Precast-GLgel Tris-Glycine PAGE (Sangon Biotech). Mouse monoclonal Myc tag antibody (60002-2-lg) was purchased from Proteintech. Rabbit monoclonal GAPDH (14C10) antibody (2118S) were purchased from Cell Signaling Technology. Mouse monoclonal SARS-CoV-2 Nucleocapsid antibody (40143-MM08) was purchased from Sino Biological. Mouse monoclonal Flag antibody (F3165) and mouse monoclonal α-tubulin antibody (T5168) was purchased from Sigma-Aldrich.

qPCR and viral RNA quantification by RT-qPCR

To detect mRNA in cells, cells were collected and the total RNA was extracted with PureLink™ RNA Mini Kit (12183018a, Thermo Fisher Scientific) according to the manufacturer's protocol. qPCR was performed using One Step TB Green® PrimeScript™ PLUS RT-PCR Kit (RR096A, Takara) with primers listed below, which were synthesized from Sangon Biotech. GAPDH was used for normalization.

Gene	Forward primer	Reverse primer
STT3A	TTGGGACGAATCATTGGAGGA	GTAAGGTGGTACGTGACGATG
STT3B	GATTCCAGCCAATCAGAACAAGT	TGCAGCTAGTGATACACCCAAA
GAPDH	GAGTCAACGGATTTGGTCGT	TTGATTTTGGAGGGATCTCG

At indicated time points after virus challenge, viral RNA in the supernatants was extracted

using the PureLink™ RNA Mini Kit according to the manufacturer's protocol. RNA quantification in each sample was performed by quantitative reverse transcription PCR (RT-qPCR) as previously reported[15].

SARS-CoV-2	Forward primer	TCCTGGTGATTCTTCTTCAGGT
	Reverse primer	TCTGAGAGAGGGTCAAGTGC
	Probe	FAM-AGCTGCAGCACCAGCTGTCCA-BHQ1
HCoV 229E	Forward primer	CGCAAGAATTCAGAACCAGAG
	Reverse primer	GGCAGTCAGGTTCTTCAACAA
	Probe	FAM-CCACACTTCAATCAAAAGCTCCCAAATG-BHQ1

RNA Interference

Caco2 cells were cultured in 24-well plates at a density of 1×10^5 per well for 18 h. siRNAs were transfected with LipofectamineTM RNAiMAX (13778150, Thermo Fisher Scientific) at a final concentration of 100 nM for twice (an interval of 48 h). 6 h after the second interference, cells were infected with viruses for 72 h. Human *STT3A* (stB0006856A), Human *STT3B* (stB0016532C) and *NC* (siN0000001) siRNAs were purchased from RiboBio.

Immunofluorescence

Cells were fixed with 4% paraformaldehyde for 15 min, permeabilized with 0.2% Triton X-100 for 10 min, and blocked in 3% BSA for 1 h. Cells were then incubated with SARS-CoV-2 Nucleocapsid antibody (40143-MM08, Sino Biological) for 1 h. Alexa Fluor FITC-conjugated secondary antibodies (ZF-0312, ZSGB-BIO) and DAPI (4083S, Cell Signaling Technology) were incubated for 1 h before the images were acquired using a IX3-RFACS fluorescence microscope (Olympus).

IC_{50} Determination

Caco2, Huh-7, HeLa-ACE2 or Vero cells were cultured in 24-well plates at a density of 2×10^5 per well for 18 h. After 1-hour infection, the medium was replaced by 2% FBS (V/V) DMEM containing NGI-1 at indicated concentrations for further 3-day treatment. Viral RNA in the supernatants was extracted and detected by RT-qPCR. Data were analyzed by GraphPad Prism 8 and IC_{50} values were calculated with SPSS (Version 21).

CC_{50} Determination

Caco2, Huh-7, HeLa-ACE2 or Vero cells were plated in a 96-well plate and cultured in the presence of NGI-1 at indicated concentrations for 3 days. The cytotoxicity was analyzed by CellTiter 96 ® AQueous One Solution Cell Proliferation Assay (G3580, Promega). The CC_{50} was calculated by SPSS and the dose-response curve was fitted by GraghPad Prism 8.

QUANTIFICATION AND STATISTICAL ANALYSIS

No statistical methods were used to estimate sample size. A standard two-tailed unpaired Student's t-test was used for statistical analysis of two groups. Statistical analyzed data are expressed as mean ± standard error of the mean (s.e.m or s.d.). A p value < 0.05 is considered as statistically significant. *$p < 0.05$, **$p < 0.01$, ****$p<0.0001$. We performed the statistical analyses using GraphPad Prism 8.

Supplementary Figures

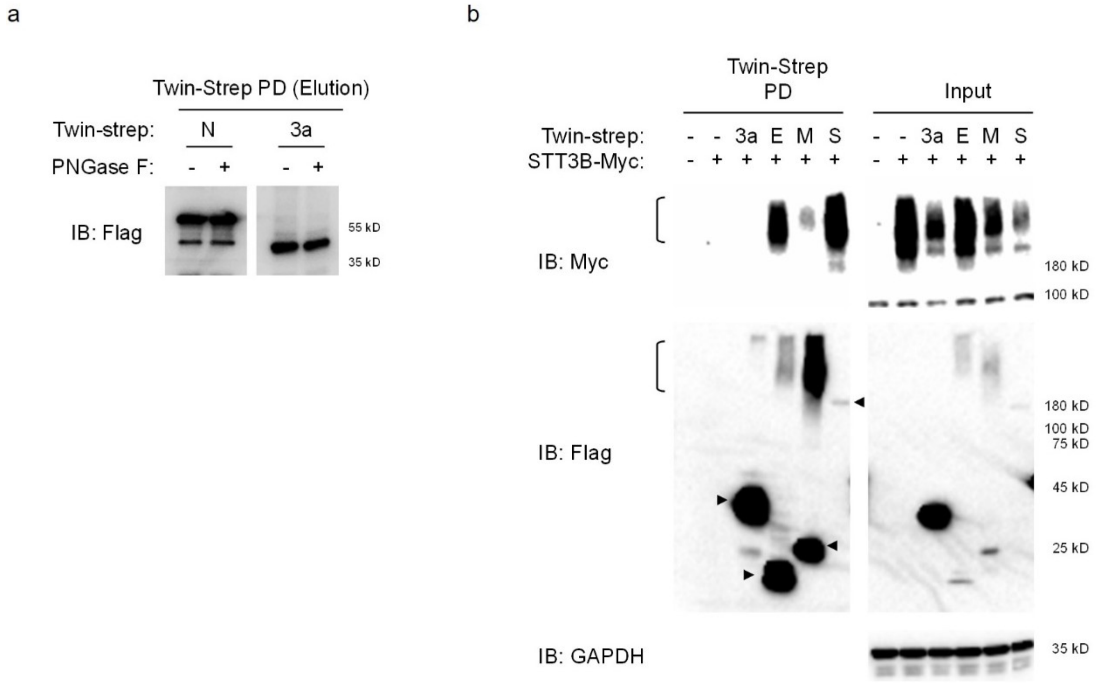

Fig.S1 Proteins of SARS-CoV-2 were interacted with STT3B.

a. 3×Flag-Twin-Strep tagged 3a or N proteins of SARS-CoV-2 were overexpressed in HEK293T. Proteins were pulled down after ultrasonication and eluted for further PNGase F treatment. Immunoblot analysis of virus proteins with Flag antibody.

b. STT3B-Myc and 3×Flag-Twin-Strep tagged 3a, E, M or S proteins of SARS-CoV-2 were overexpressed in HEK293T, followed by Twin-Strep pull-down. STT3B was analyzed with Myc antibody and virus proteins were analyzed with Flag antibody. The anti-GAPDH blots indicated loading of lanes.

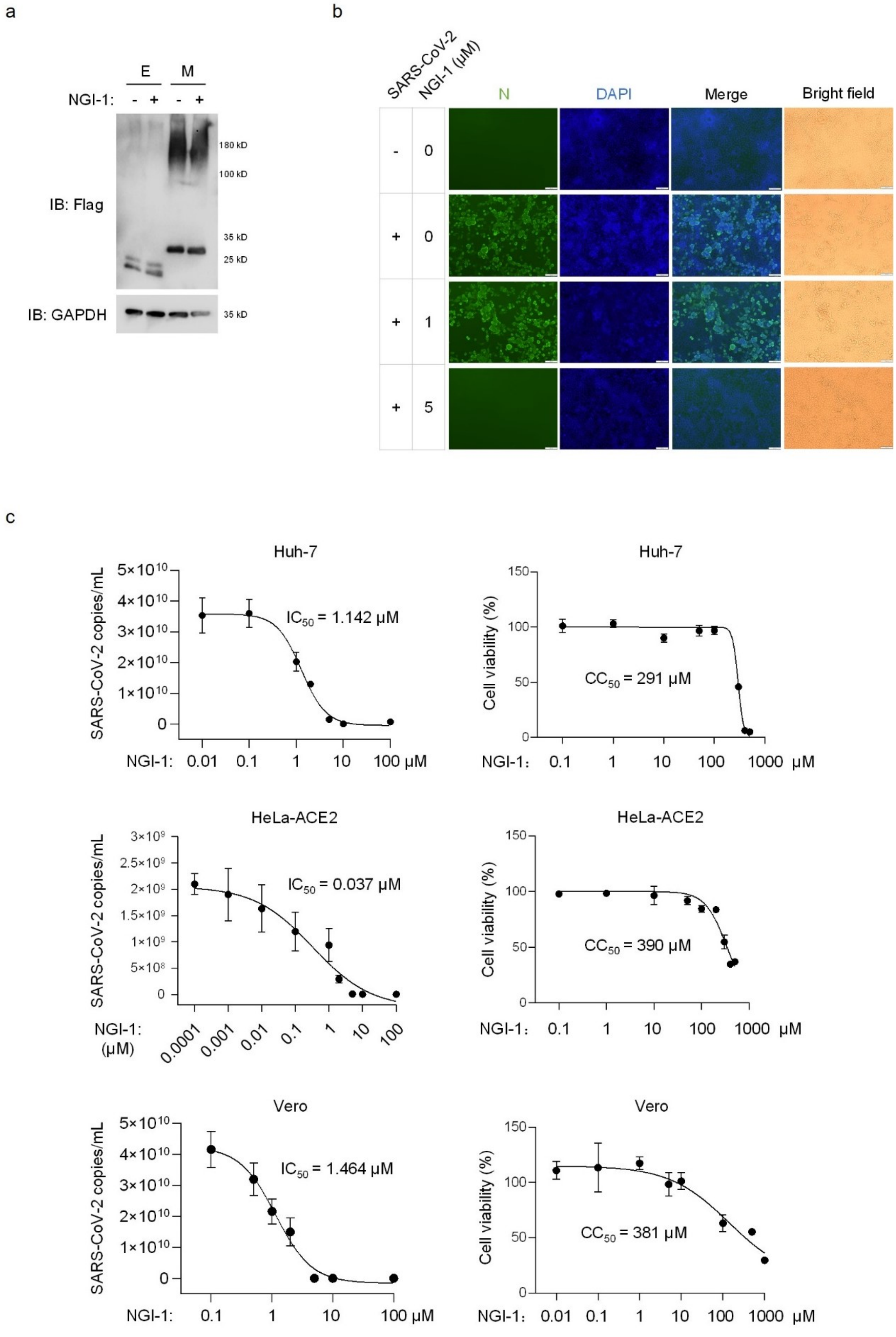
a
E
M
NGI-1:
IB: Flag
IB: GAPDH
180 kD
100 kD
35 kD
25 kD
35 kD
b
SARS-CoV-2
NGI-1 (μM)
N
DAPI
Merge
Bright field
c
Huh-7
IC50 = 1.142 μM
SARS-CoV-2 copies/mL
NGI-1:
CC50 = 291 μM
Cell viability (%)
HeLa-ACE2
IC50 = 0.037 μM
CC50 = 390 μM
Vero
IC50 = 1.464 μM
CC50 = 381 μM

Fig.S2 NGI-1 inhibited SARS-CoV-2.

a. 3×Flag tagged E or M proteins of SARS-CoV-2 were overexpressed in HEK293T, followed by 5 μM NGI-1 treatment for 2 days. Whole cell lysates were collected for Immunoblot analysis. Virus proteins were analyzed with Flag antibody. The anti-GAPDH blots indicated loading of lanes.

b. Caco2 cells were infected with SARS-CoV-2 (MOI = 0.01) for 1 hour, and the medium was replaced by 2% FBS (V/V) DMEM containing 0, 1, or 5 μM NGI-1 for further 3-day treatment. Virus in cells was detected by immunofluorescence with N protein antibody. DAPI (blue) stained the nuclei and the bright field showed the cell morphology. Scale bar, 100 μm.

c. Huh-7, HeLa-ACE2 or Vero cells were infected with SARS-CoV-2 (MOI = 0.01) for 1 hour, followed by treatment of NGI-1 at the indicated concentrations for 3 days. Virus in the supernatant was analyzed by RT-qPCR. For CC_{50} detection, cells were plated in a 96-well plate and cultured in the presence of NGI-1 at indicated concentrations for 3 days. The cytotoxicity was analyzed by CellTiter 96 ® AQueous One Solution Cell Proliferation Assay. The IC_{50} and CC_{50} was calculated by SPSS and the dose-response curve was fitted by GraghPad Prism 8.

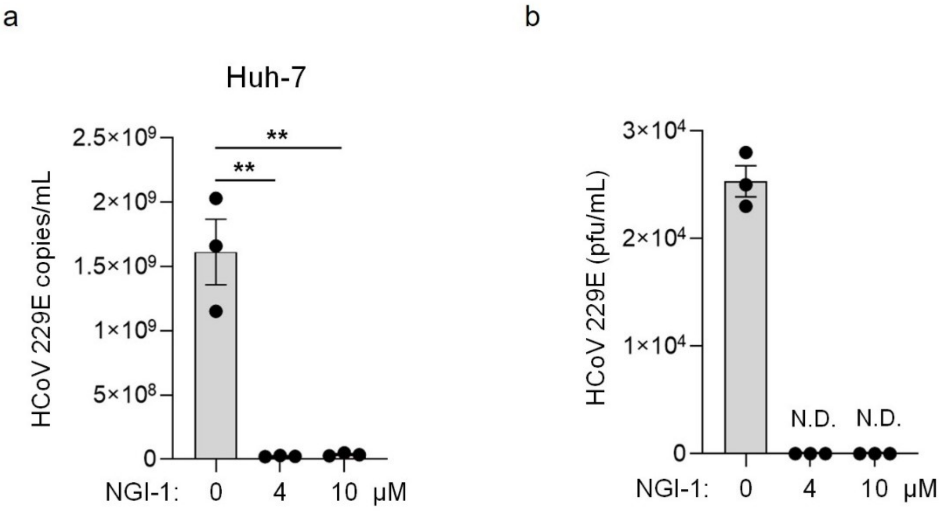

Fig.S3 NGI-1 inhibited HCoV 229E.

a-b. Huh7 cells were infected with HCoV 229E (MOI = 0.1) for 1 hour, followed by treatment of NGI-1 at the indicated concentrations for 3 days. Virus copies in the supernatant was analyzed by RT-qPCR (**a**). The titer of HCoV 229E in the supernatant was analyzed in Huh7 cells (**b**). N.D. indicated non-detected. Data are mean ± SEM, from triplicates (biological replicates), unpaired t test, $^{**}p < 0.01$.

Supplementary reference

15 Zhang, N. N. *et al.* A Thermostable mRNA Vaccine against COVID-19. *Cell* **182**, 1271-1283 e1216 (2020).
